# The Effects of Methylphenidate on Resting-State Functional Connectivity of the Basal Nucleus of Meynert, Locus Coeruleus, and Ventral Tegmental Area in Healthy Adults

**DOI:** 10.3389/fnhum.2016.00149

**Published:** 2016-04-18

**Authors:** Ryan L. Kline, Sheng Zhang, Olivia M. Farr, Sien Hu, Laszlo Zaborszky, Gregory R. Samanez-Larkin, Chiang-Shan R. Li

**Affiliations:** ^1^Department of Psychology, Yale University School of Arts and SciencesNew Haven, CT, USA; ^2^Department of Psychiatry, Yale University School of MedicineNew Haven, CT, USA; ^3^Interdepartmental Neuroscience Program, Yale UniversityNew Haven, CT, USA; ^4^Center for Molecular and Behavioral NeuroscienceRutgers, NJ, USA; ^5^Department of Neurobiology, Yale University School of MedicineNew Haven, CT, USA

**Keywords:** resting state connectivity, BOLD, basal forebrain, midbrain, acetylcholine, dopamine, norephinephrine

## Abstract

**Background:** Methylphenidate (MPH) influences catecholaminergic signaling. Extant work examined the effects of MPH on the neural circuits of attention and cognitive control, but few studies have investigated the effect of MPH on the brain's resting-state functional connectivity (rsFC).

**Methods:** In this observational study, we compared rsFC of a group of 24 healthy adults who were administered an oral 45 mg dose of MPH with a group of 24 age and gender matched controls who did not receive MPH. We focused on three seed regions: basal nucleus of Meynert (BNM), locus coeruleus (LC), and ventral tegmental area/substantia nigra, pars compacta (VTA/SNc), each providing cholinergic, noradrenergic and dopaminergic inputs to the cerebral cortex. Images were pre-processed and analyzed as in our recent work (Li et al., 2014; Zhang et al., 2015). We used one-sample *t*-test to characterize group-specific rsFC of each seed region and two-sample *t*-test to compare rsFC between groups.

**Results:** MPH reversed negative connectivity between BNM and precentral gyri. MPH reduced positive connectivity between LC and cerebellum, and induced positive connectivity between LC and right hippocampus. MPH decreased positive VTA/SNc connectivity to the cerebellum and putamen, and reduced negative connectivity to left middle occipital gyrus.

**Conclusion:** MPH had distinct effects on the rsFC of BNM, LC, and VTA/SNc in healthy adults. These new findings may further our understanding of the role of catecholaminergic signaling in Attention Deficit Hyperactivity Disorder (ADHD) and Parkinson's disease and provide insights into the therapeutic mechanisms of MPH in the treatment of clinical conditions that implicate catecholaminergic dysfunction.

## Introduction

We seek to characterize the effects of methylphenidate (MPH) on resting state functional connectivity (rsFC) in humans. This study expands upon a previous work, which focused on the dorsal striatum and thalamus (Farr et al., 2014a), by describing the effects of MPH on the whole-brain rsFC of the basal nucleus of Meynert (BNM), locus coeruleus (LC), and the ventral tegmental area/substantia nigra pars compacta (VTA/SNc), each providing cholinergic, noradrenergic (NA), and dopaminergic (DA) inputs, respectively, to the cerebral cortex.

Low frequency, “spontaneous” blood oxygenation level dependent (BOLD) signals are spatially organized and provide valuable insights to the functional architecture of the brain (Fair et al., 2007; Fox and Raichle, 2007). Brain regions involved in similar tasks show correlated BOLD responses during rest. This includes functional connectivity between regions associated with sensorimotor processing, language, visual perception (Cordes et al., 2000), memory (Vincent et al., 2006), and attention (Fox et al., 2006a). A simple, sensitive measure of coordinated regional brain activations, rsFC examines how individual voxels are functionally related by increasing or decreasing activity concurrently (Fox and Raichle, 2007). Using this method, we previously delineated functional subdivisions in the medial superior frontal cortex (Zhang et al., 2012), precuneus (Zhang and Li, 2012) and the inferior parietal lobule (Zhang and Li, 2014), and characterized whole-brain functional connectivity of the BNM and VS (Li et al., 2014) as well as the LC and VTA/SNc (Zhang et al., 2015).

The current study focuses on subcortical nuclei that mediate cholinergic and catecholaminergic signaling as these circuits are of great importance to both basic and clinical neuroscience. For instance, although MPH has been used to treat ADHD and other clinical conditions since the 1950s (Lange et al., 2010), research into its mechanism of action and effects on brain function has only recently begun (for review, see Solanto, 1998; Advokat, 2010; Sahakian et al., 2015). We describe the rationale of the study by focusing on the functional anatomy of the cholinergic and catecholaminergic systems and how these neurotransmitter systems are implicated in the pathophysiology of neuropsychiatric conditions.

### Basal nucleus of meynert (BNM)

The BNM provides cholinergic inputs to the hippocampus, olfactory bulb, amygdala, and all of the neo-cortex (Pearson et al., 1983; Rye et al., 1984; Richardson and DeLong, 1986). Functionally, BNM is associated with memory formation (Richardson and DeLong, 1988), attention, and the regulation of arousal and sleep (Wenk, 1997). Selective inhibition of BNM leads to memory deficits in rats (Voytko et al., 1994; Stoehr et al., 1997; Tian et al., 2004), which are reversible with a cholinergic agonist (Ridley et al., 1986) or catecholamine enzyme inhibitor, suggesting an interaction between the catecholaminergic and cholinergic systems (Khromova et al., 1995).

40% to 76% of BNM neurons are lost (Tagliavini and Pilleri, 1983) and choline acetyltransferase is diminished by 90% (Candy et al., 1983) in Alzheimer's disease (AD). Given the efficacy of cholinergic treatments (Wilson et al., 1995; Bodick et al., 1997; Rogers et al., 1998; Tariot et al., 2000), the loss of BNM volume likely underlies cognitive dysfunction in AD. Patients with Parkinson's disease (PD) demonstrated a concurrent decrease in choline acetyltransferase in the neocortex and in the number of BNM neurons (Perry et al., 1985). Further, studies have linked cholinergic deficits to catecholaminergic dysfunction in these degenerative conditions. In AD, acetylcholine depletion correlates with NA (Yates et al., 1983) as well as DA and serotoninergic (Reinikainen et al., 1990) depletion.

The interaction of catecholaminergic and cholinergic systems is also evident in other animal studies (Janowsky et al., 1974; Tellez et al., 1999; Cucchiaro and Commons, 2003). Rats treated with MPH exhibit stereotyped gnawing behavior, which can be reduced with physostigmine, a cholinesterase inhibitor, both pre- and post-treatment (Janowksy et al., 1972). A similar antagonistic relationship between the two drugs was found in human schizophrenia and mania patients, with physostigmine and MPH each enhancing and impeding behavioral inhibition (Janowsky et al., 1973). While it is not clear where in the neural circuits these interactions transpire, the BNM receives direct projections from the VTA/SNc (Gaykema and Zaborszky, 1996) and modulates nigrostriatal circuit activity (Haber and Knutson, 2009). It is plausible that the DA and cholinergic systems have cascading interactions at multiple levels of representations. By addressing the effect of MPH on the cerebral functional connectivity of the BNM, the present study would provide some information on this issue.

### Locus coeruleus (LC)

LC is the largest source of NA neurons in the central nervous system (CNS) (Moore and Bloom, 1979; Foote et al., 1983), supporting arousal and cognitive functioning (Berridge and Waterhouse, 2003; Aston-Jones and Cohen, 2005). Phasic LC activation quickly follows presentation of target stimuli, precedes the delivery of associated reward, and expresses an anticipation signal (Aston-Jones et al., 1985, 1994), with concurrent norepinephrine (NE) release in the cortex (Mountcastle et al., 1972; Aston-Jones and Cohen, 2005). This phasic pattern of activation resembles that found in the DA systems (Schultz et al., 1997). In contrast, tonic baseline activity of the LC corresponds with less efficient task-related behavior in animals (Aston-Jones and Cohen, 2005) and may support exploration in situations where the value of the task at hand has declined (Usher et al., 1999). Projections to the LC from the orbitofrontal cortex (OFC) and anterior cingulate cortex (ACC), areas critical to decision-making (O'Doherty et al., 2003; Hare et al., 2008), may facilitate switching between tonic and phasic modes of operation. MPH increases NE levels in the LC and indeed throughout the brain (Gatley et al., 1996; Kuczenski and Segal, 1997, 2002; Hannestad et al., 2010). MPH reduces both tonic and phasic LC firing in a dose-dependent manner, with more prominent effects at high doses and a stronger effect on phasic than on tonic activity at low doses (Devilbiss and Berridge, 2006).

MPH ameliorates impulsivity both in humans and non-human primates (Rajala et al., 2012, 2015; Berridge and Arnsten, 2013). Thus, examining the effects of MPH on LC connectivity will advance our understanding of its neural mechanisms in impulse control and MPH's therapeutic effects in ADHD (Volkow et al., 1995). Numerous studies revealed an important role of NE in cognition. NA α-1-adrenoceptor antagonist impairs working memory, particularly during induced stress (Birnbaum et al., 1999). NA depletion impaired prefrontal cortical function, which could be restored by NA α-2 agonists (Arnsten et al., 1996). NA dysfunction is implicated in cognitive deficits in Down syndrome (Salehi et al., 2009), schizophrenia, and AD (Friedman et al., 1999). NE also plays a role in affective memory; β-adrenoceptor antagonists impair memory of emotional stimuli (Cahill et al., 1994). NE agents have been widely used in the treatment of depression, with newer medications increasing NE levels along with serotonin and/or dopamine (Nelson et al., 2004; Stahl et al., 2004; Blier and Szabo, 2005; Joffe et al., 2007). Thus, understanding the rsFC of the LC would help advance our knowledge of the cerebral NA system and its relevance to clinical conditions other than ADHD.

### Ventral tegmental area/substantia nigra, pars compacta (VTA/SNc)

The VTA/SNc projects to the striatum and neocortex and receives heavy glutamatergic projections from the ventromedial prefrontal cortex (vmPFC), OFC, dorsal ACC (dACC), as well as the hippocampus and amygdala (Haber and Knutson, 2009).

The DA pathway is a major component of the reward system, a network of brain regions that predict and encode value during reward-based processing and learning (Schultz et al., 1997; O'Doherty et al., 2002; McClure et al., 2003; Aron et al., 2005; Haber et al., 2006; D'Ardenne et al., 2008; Haber and Knutson, 2009). Stimulants including MPH, atomoxetine (ATX), and amphetamines influence glutamatergic signaling of DA neurons in the VTA (Kalivas and Weber, 1988; Pert, 1998), with repeated exposure leading to behavioral sensitization to stimulants (Bonci and Williams, 1996; Pierce and Kalivas, 1997), as also observed in humans (Prieto-Gómez et al., 2005; Jones and Dafny, 2013). Psychostimulants may impact the brain via connectivities to the VTA, with dysfunctional changes leading to addiction.

MPH elicited increase in BOLD activity in the SN of rats (Easton et al., 2009). In an arterial spin labeling study of humans, MPH and ATX respectively increased and decreased regional cerebral blood flow to the SN/midbrain (Marquand et al., 2012). MPH also influences VTA/SN activation to behavioral tasks, reversing responses to mental fatigue for both healthy controls and cocaine abusing individuals (Moeller et al., 2012). MPH blocks more than 50% of dopamine transporter and significantly increases levels of extracellular DA in the basal ganglia (Volkow et al., 1995, 1998, 2002). In fMRI, boys with ADHD show increased, while healthy controls show decreased, activity in the striatum under MPH (Vaidya et al., 1998). MPH improves ADHD symptoms by increasing frontal and striato-thalamic activation for inhibitory control (Rubia et al., 2011), but it is unclear whether these effects are mediated by DA projections from VTA (Shen and Choong, 2006; Warton et al., 2009). Examining the influence of MPH on cerebral functional connectivity of VTA/SNc will provide useful information on this issue.

## Methods

Participants, study procedures, and imaging pre-processing were described in detail in our recent work (Farr et al., 2014a).

### Participants

Twenty-four healthy adults (16 females; age 25 ± 6 years) participated in the study. All were without medical, neurological, or psychiatric conditions, denied history of head injury and current use of prescription medications or illicit substances, and showed negative urinalysis on the day of fMRI. These 24 participants received a single 45 mg oral dose of MPH before fMRI and comprised the methylphenidate (MPH) group. Data of a cohort of 24 matched healthy participants (16 females; age 24 ± 4 years) scanned under identical imaging protocols except without being given MPH were used for comparison—the no-MPH group. Compared to baseline, MPH increased heart rate, systolic blood pressure, and anxiety rating, as we reported recently (Farr et al., 2014b). All participants provided written consent following a protocol approved by the Yale Human Investigation Committee.

### Imaging protocol and data analysis

Conventional T1-weighted spin-echo sagittal anatomical images were acquired for slice localization using a 3T scanner (Siemens Trio). Anatomical images of the functional slice locations were next obtained with spin-echo imaging in the axial plane parallel to the AC-PC line with TR = 300 ms, TE = 2.5 ms, bandwidth = 300 Hz/pixel, flip angle = 60°, field of view = 220 × 220 mm, matrix = 256 × 256, 32 slices with slice thickness = 4 mm and no gap. Functional, BOLD signals were then acquired with a single-shot gradient echo echo-planar imaging (EPI) sequence. Thirty-two axial slices parallel to the AC-PC line covering the whole brain were acquired with repetition time = 2000 ms, echo time = 25 ms, bandwidth = 2004 Hz/pixel, flip angle = 85°, field of view = 220 × 220 mm, matrix = 64 × 64, 32 slices with slice thickness = 4 mm and no gap. Three hundred images were acquired in the resting state during which participants were instructed to close their eyes but stay awake for a period of 10 min (Farr et al., 2014b).

#### Imaging data pre-processing

Brain imaging data were pre-processed using the same routine as described in our previous work (Zhang et al., 2012). Briefly, images of each individual subject were first realigned (motion corrected) and corrected for slice timing. A mean functional image volume was constructed for each subject from the realigned image volumes. The high-resolution structural image was co-registered with these mean images and then segmented for normalization with affine registration followed by nonlinear transformation (Friston et al., 1995; Ashburner and Friston, 1999). The normalization parameters determined for the structural volume were then applied to the corresponding functional image volumes for each subject. Finally, the images were smoothed with a Gaussian kernel of 8 mm at full width at half maximum.

Additional pre-processing was applied to reduce spurious BOLD variances that were unlikely to reflect neuronal activity (Rombouts et al., 2003; Fox et al., 2006a; Fair et al., 2007; Fox and Raichle, 2007). The sources of spurious variance were removed through linear regression by including the signal from the ventricular system, the white matter and the whole brain, in addition to the six parameters obtained by rigid body head motion correction. First-order derivatives of the whole brain, ventricular, and white matter signals were also included in the regression. Following earlier studies (Cordes et al., 2001; Fox and Raichle, 2007), we applied a temporal band-pass filter (0.009 Hz < f < 0.08 Hz) to the time course in order to obtain low-frequency fluctuations (Fox et al., 2006b; Fair et al., 2007; Fox and Raichle, 2007).

As extensively investigated by Van Dijk et al. (2012), we applied a “scrubbing” method proposed by Power and colleagues (Smyser et al., 2010; Power et al., 2012; Tomasi and Volkow, 2014) to remove time points affected by head motions: for every time point *t*, we computed the *framewise displacement* given by *FD*(*t*) = |Δ*d*_*x*_(*t*)| + |Δ*d*_*y*_(*t*)| + |Δ*d*_*z*_(*t*)| +*r*|α(*t*)| +*r*|β(*t*)| +*r*|γ(*t*)|, where (*d*_*x*_, *d*_*y*_, *d*_*z*_) and (α, β, γ) are the translational and rotational movements, respectively, and the root mean square variance (DVARS) of the differences in % BOLD intensity *I*(*t*) between consecutive time points across brain voxels: $DVARS\left(t\right)=\sqrt{\langle |I\left(t\right)-I\left(t-1\right){|}^{2}\rangle }$. To compute each subject's correlation map, we removed every time point that exceeded the head motion limit *FD*(*t*) > 0.5 mm or DVARS(*t*) > 0.5% (Power et al., 2012; Tomasi and Volkow, 2014). On average, 1% of the time points were removed across subjects.

#### Seed regions

**Figure 1:**
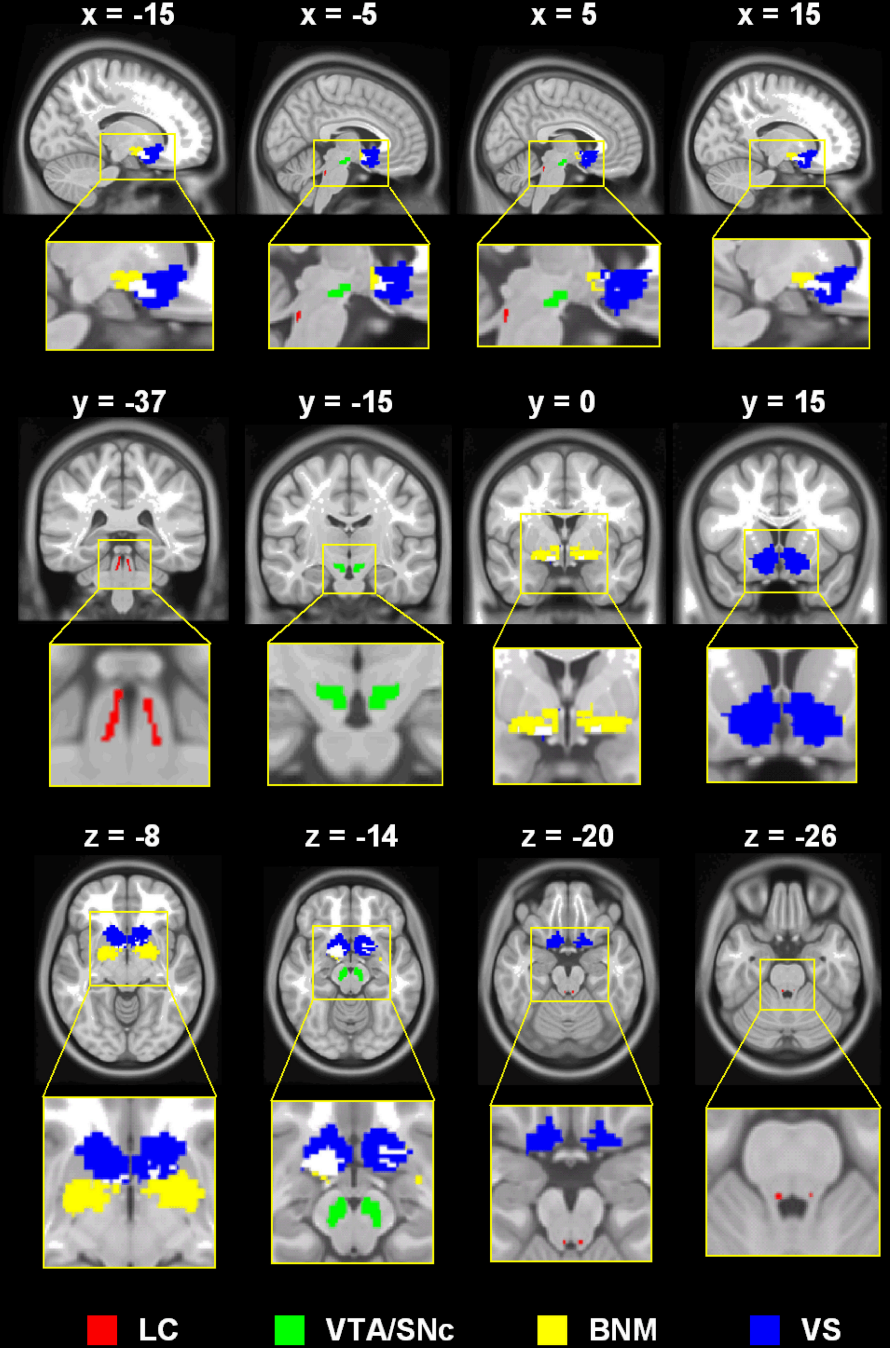
**Seed regions: LC, locus coeruleus; VTA/SNc, ventral tegmental area, pars compacta; BNM, basal nucleus of meynert; VS, ventral striatum (shown for contrast with the BNM; not examined in the current work)**.

We used the same seed regions as in our earlier work (Li et al., 2014; Manza et al., 2015; Zhang et al., 2015), which are shown in Figure 1.

##### Basal nucleus of meynert (BNM)

A mask of the BNM was created based on a stereotaxic probabilistic map of magnocellular cell groups in the basal forebrain (Zaborszky et al., 2008), as detailed in our earlier work (Li et al., 2014). Briefly, a T1-weighted MRI scan of 1.17 × 1 × 1 mm was obtained of each individual brain (*n* = 10) before histological processing. The outlines of various basal forebrain compartments were traced on 2D images of silver-stained (Merker, 1983) histological sections (20 μm thick, 1.2 mm apart) with a resolution of 7000 × 6000 pixels. The outlines were processed as contour line for each histological section. As described in Zaborszky et al. (2008), we used a modified version of the Ch1–Ch4 nomenclature of Mesulam et al. (1983) to delineate the magnocellular basal forebrain cell groups. Cell aggregates in the subcommissural–sublenticular region largely correspond to the BNM as defined by Mesulam et al. (1983); Vogels et al. (1990); De Lacalle et al. (1991); Zaborszky et al. (2008).

##### Locus coeruleus (LC) and ventral tegmental area/substantia nigra, pars compacta (VTA/SNc)

We used a probabilistic template of the LC derived by Keren et al. (2009). The LC seed region represents the extent of peak LC signal distribution, obtained from a sample of 44 healthy adults (age range: 19–79 years) using high-resolution T1-weighted Turbo Spin Echo (T1-TSE) MRI, and has a volume of 93 mm^3^. The T1-TSE LC signals were likely influenced by the ferrous neuromelanin metabolites within LC neurons (Sasaki et al., 2006) and observed in sections corresponding to the greatest concentrations of LC cells in postmortem studies (German et al., 1988). The VTA/SNc region was derived from the structural MRIs of 30 healthy adults; after spatial normalization and averaging across subjects, the size of the bilateral mask was 1106 mm^3^ (Ahsan et al., 2007).

#### Seed-based functional connectivity: linear correlations

The BOLD time courses were averaged spatially across all voxels each for the three seed regions. We computed the correlation coefficient between the averaged time course of each mask and the time courses of individual voxels of the brain for individual subjects. To assess and compare the resting state “correlograms,” we converted these image maps, which were not normally distributed, to z score maps by Fisher's z transform (Jenkins and Watts, 1968; Berry and Mielke Jr, 2000): z = 0.5 loge[(1+r)/(1−r)]. The z maps were used in group random effect analyses (Penny et al., 2004) with a two- sample *t*-test to compare MPH and no-MPH groups. All imaging findings were examined with a peak voxel *p* < 0.001, uncorrected, combined with a cluster threshold *p* < 0.05, corrected for family-wise error of multiple comparisons.

## Results

**Figure 2:**
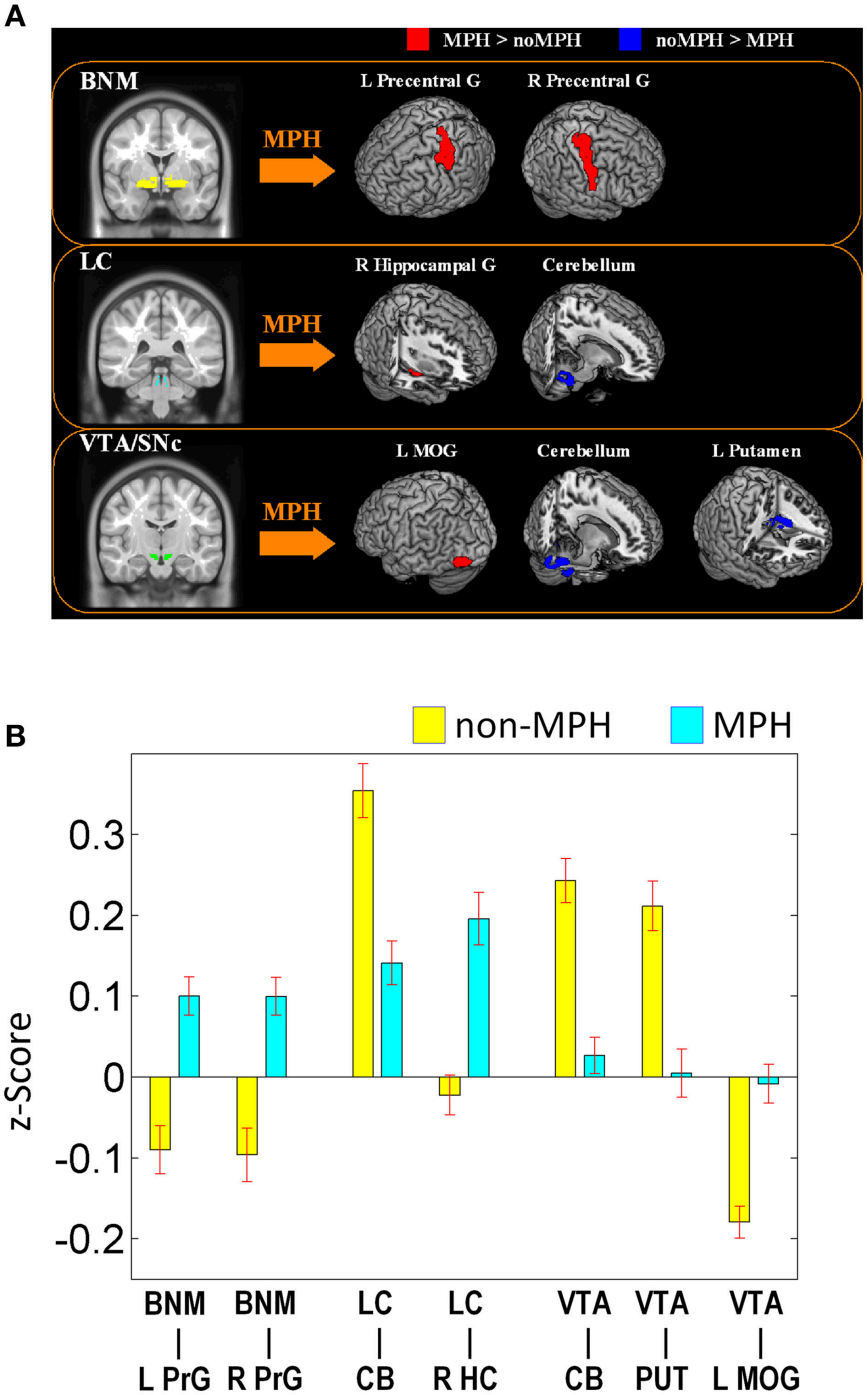
**(A)** Brain regions that show different connectivities between MPH and noMPH groups; **(B)** Effect size (*z*-value) of rsFC of brain regions that show different connectivities between MPH and noMPH groups. Values are mean ± standard error; BNM, basal nucleus of Meynert; LC, locus coeruleus; VTA/SNc, ventral tegmental area/substantia nigra pars compacta; PrG, precentral gyrus; MOG, middle occipital gyrus; HC, hippocampus; MOG, middle occipital gyrus; CB, cerebellum; PUT, putamen.

The main results of the differences in rsFC between the two groups are summarized in Figure 2 and Table 1.

**Table 1:** **Brain regions that show different connectivities between MPH and noMPH groups**.

**Cluster size (mm^3^)**	**Voxel *Z*-value**	**MNI coordinate (mm)**			**Identified region**
		***x***	***y***	***z***	
**BNM CONNECTIVITY: MPH** > **noMPH**					
6507	4.20	−36	−28	70	L Precentral gyrus
	4.10	−51	−22	58	
	4.09	−24	−31	76	
7992	4.14	30	−31	70	R Precentral gyrus
	3.85	48	−16	64	
	3.78	51	−16	43	
**BNM CONNECTIVITY: noMPH** > **MPH**					
None					
**LC CONNECTIVITY: MPH** > **noMPH**					
2484	4.71	27	−16	−17	R Hippocampal gyrus
	3.44	15	−10	−23	
	3.43	36	−31	−5	
**LC CONNECTIVITY: noMPH** > **MPH**					
5373	4.70	12	−43	−50	R Cerebellum
	3.90	9	−52	−50	
	3.67	−15	−46	−47	L Cerebellum
**VTA/SNc CONNECTIVITY: MPH** > **noMPH**					
3537	4.40	−30	−82	−14	L Middle occipital gyrus
	4.22	−48	−79	−5	
	3.36	−18	−88	−11	
**VTA/SNc CONNECTIVITY: noMPH** > **MPH**					
24,975	4.55	15	−52	−35	R Cerebellum
	4.46	3	−73	−17	
	4.42	12	−82	−32	
4104	4.24	−21	−1	13	L Putamen/Pallidum
	3.76	−9	−13	13	

*All peaks greater than 8 mm apart are identified. One voxel is 3 × 3 × 3 mm^3^*.

*BNM, basal nucleus of Meynert; VS, ventral striatum; LC, locus coeruleus; VTA/SNc, ventral tegmental area, pars compacta; p < 0.001 uncorrected and cluster-level threshold of p < 0.05, FWE corrected*.

### Basal nucleus of meynert

**Figure 3:**
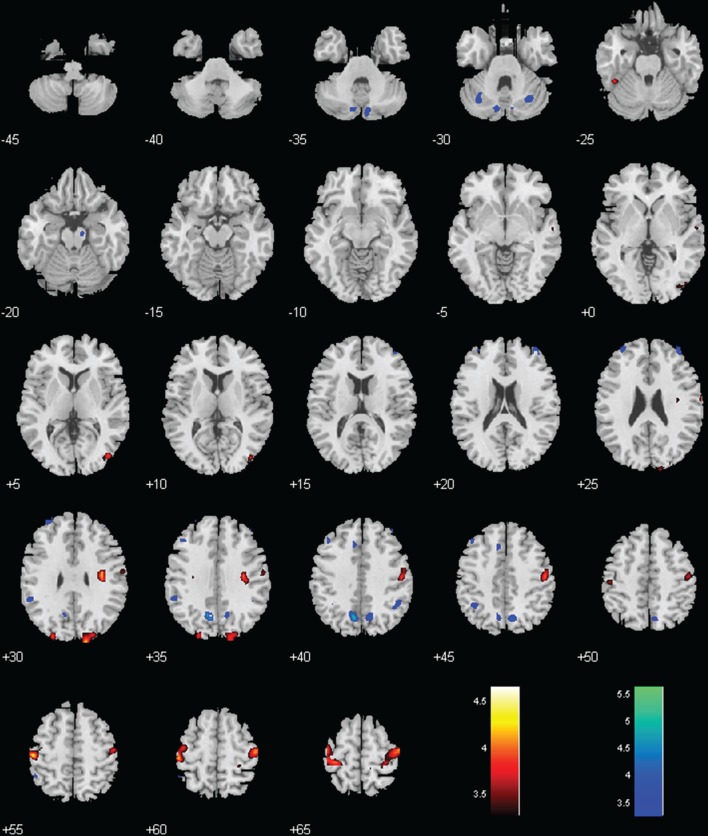
**Regions with different functional connectivity to the BNM; Warm colors: MPH > no-MPH; Cool colors: MPH < no-MPH; voxel *p* < 0.001 uncorrected and cluster *p* < 0.05, FWE corrected**. Color bars represent voxel *T*-value.

MPH reversed negative connectivity between the BNM and bilateral precentral gyri, including regions of the primary motor and premotor cortex (Figures 2, 3; Table 1).

### Locus coeruleus

**Figure 4:**
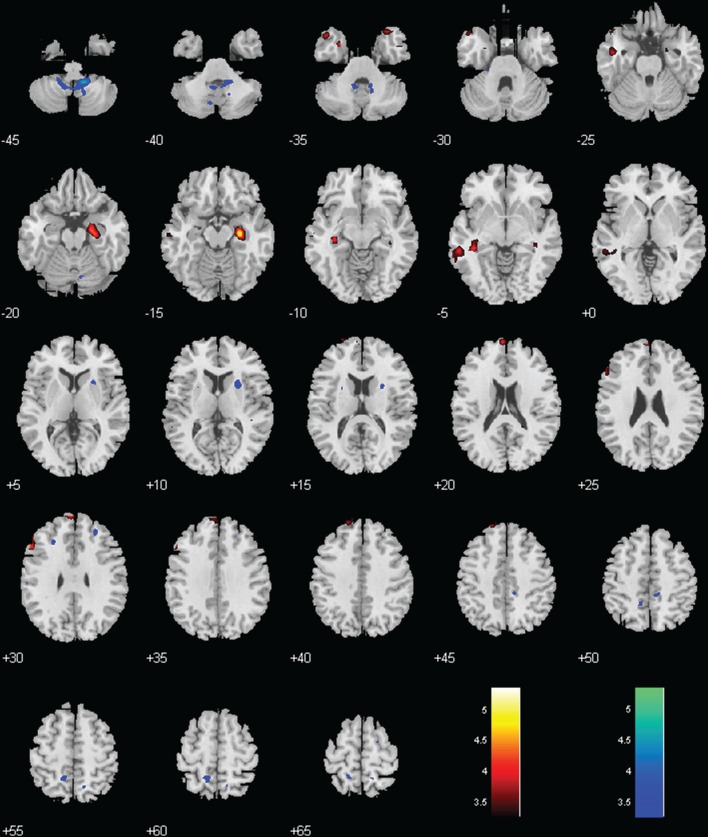
**Regions with different functional connectivity to the LC**. Warm colors: MPH > no-MPH; Cool colors: MPH < no-MPH; voxel *p* < 0.001 uncorrected; and cluster *p* < 0.05, FWE corrected. Color bars represent voxel *T*-value.

Both groups showed positive connectivity of LC with the bilateral cerebellum, with the MPH group showing significantly less positive connectivity. While the no-MPH group showed no significant connectivity of LC with the right hippocampus, the MPH group showed significant positive connectivity (Figures 2, 4; Table 1).

### Ventral tegmental area/substantia nigra, pars compacta (VTA/SNc)

**Figure 5:**
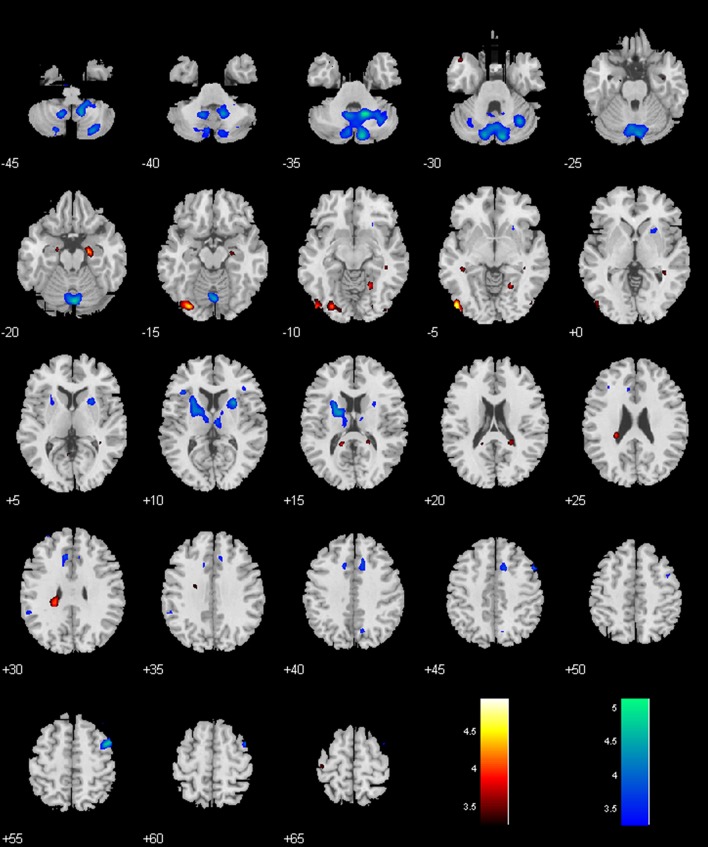
**Regions with different functional connectivity to the VTA/SNc**. Warm colors: MPH > no-MPH; Cool colors: MPH < no-MPH; voxel *p* < 0.001 uncorrected; and cluster *p* < 0.05, FWE corrected. Color bars represent voxel *T*-value.

While the no-MPH group showed negative VTA/SNc connectivity with the left middle occipital gyrus (MOG), the MPH group showed no significant connectivity. Both groups showed positive VTA/SNc connectivity with bilateral cerebellum, with the MPH group showing lower connectivity than the no-MPH group. While the no-MPH group showed positive connectivity with bilateral putamen the MPH group showed no significant connectivity (Figures 2, 5; Table 1).

### Analyses without global signal regression in data pre-processing

We re-analyzed the data without using global signal regression in pre-processing. The findings showed that changes in functional connectivity were slightly diminished in significance but were otherwise similar. To confirm the findings, we extracted the effect size of connectivity (in data without global signal regression) of the ROIs as identified from the original analysis for comparison between the MPH and no-MPH groups (Figure 2). The results showed that the effect size of connectivity difference remained highly significant: BNM—L Precentral Gyrus (*p* < 0.00001); BNM—R Precentral Gyrus (*p* < 0.00002); LC—Cerebellum (*p* < 0.000001); LC—Hippocampus (*p* < 0.0004); VTA/SNc—Cerebellum (*p* < 0.000001); VTA/SNc—Putamen (*p* < 0.0012); and VTA/SNc—Left MOG (*p* < 0.0010).

## Discussions

### Basal nucleus of meynert

Anatomically, VTA/SNc projects directly to the BNM (Gaykema and Zaborszky, 1996). The dopamine-beta-hydroxylase and tyrosine-hydroxylase containing axons meet the cholinergic neurons, suggesting catecholaminergic modulation of cholinergic activity (Zaborsky and Cullinan, 1996). BNM provides cholinergic inputs to most of the cerebral cortex, including the primary motor cortex (PMC; Pearson et al., 1983) and receives projections from the OFC and VS (Mesulam and Mufson, 1984), both of which are targets of DA projections and implicated in reward prediction, valuation, and decision-making (Haber et al., 2006; Haber and Knutson, 2009). Thus, MPH-induced increases in functional connectivity between BNM and motor cortex may be mediated directly by VTA/SNc DA projections or indirectly via the OFC and VS.

MPH's effects on the motor systems are well-documented. MPH increased frontal activation in both healthy and ADHD children (Vaidya et al., 1998). MPH increased the intensity and decreased inhibition of motor responses in studies of transcranial magnetic stimulation in both healthy individuals (Ilic et al., 2003) and ADHD patients (Gilbert et al., 2005). MPH also increased activity in lateral premotor areas in a four-choice reaction time task (Müller et al., 2005). Additionally, basal forebrain cholinergic outputs are required for motor learning. Lesions of the rodent homolog of BNM depleted cholinergic innervation to the cortex by more than 99% and led to slower and less accurate responses in motor learning, but not in performing a previously learned motor task (Conner et al., 2003, 2005, 2010). Together, BNM and its cholinergic outputs to the motor cortex are essential for motor control and learning, and MPH may influence this process by reversing the negative BNM—motor cortical connectivity.

MPH elicited reversal of the sign of BNM—motor cortical connectivity may have treatment implications for PD. Combined with levodopa (L-Dopa), MPH improves hand tapping speed but worsens dyskinesia symptoms as compared to L-Dopa alone (Camicioli et al., 2001). MPH alone improved reaction time in a choice task (Camicioli et al., 2001) but may improve (Devos et al., 2007; Moreau et al., 2012) or worsen (Espay et al., 2011) gait symptoms in PD patients. These complexities need to be resolved in the future.

### Locus coeruleus

MPH significantly reduced positive LC connectivity with specific regions of the cerebellar cortex, likely lobules IV, V, and X (Schmahmann et al., 2000). LC is the primary source of NA projections to the cerebellum and cerebral cortex (Berridge and Waterhouse, 2003). LC projects to the cerebellum via the superior cerebellar peduncle (Moore and Bloom, 1979) and synapses with inhibitory Purkinje cell dendrites in the Purkinje and molecular layers (Ito et al., 1964; Hoffer et al., 1973; Moises and Woodward, 1980). Thus, MPH may alter activities of inhibitory Purkinje cells in the cerebellum. Given that areas of the cerebellum are specialized for fine-tuning of motor control and/or calibration of motor output (Middleton and Strick, 2000), it is possible that MPH's effect on the reduction of positive connectivity represents a shift away from fine in favor of gross motor function.

It has been suggested that long-term potentiation (LTP) in the hippocampus is mediated by NA activity (Ramos and Arnsten, 2007). Increases in NE, as elicited by an alpha-1-adrenergic agonist, significantly increased LTP in the hippocampus (Izumi and Zorumski, 1999). MPH increases NE levels (Weikop et al., 2007) and enhances both LTP and long-term depression in the hippocampus, both of which were blocked by a beta-noradrenoceptor antagonist (Dommett et al., 2008). Thus, MPH-evoked increases in LC connectivity to the hippocampus may alter learning and memory.

Indeed, MPH improved 1-week retention of both casually and intentionally learned information when administered 12 h after learning (Izquierdo et al., 2008). When administered before testing, MPH improved word recall, but not spatial working memory in healthy adults (Verster et al., 2010; Linssen et al., 2012). In contrast, MPH appears to reduce emotional memory despite producing increased arousal for emotional stimuli (Brignell et al., 2007). Polymorphisms of Catechol-O-methyltransferase (COMT) gene have been associated with different degrees of cognitive improvements under amphetamine (Mattay et al., 2003), highlighting inter-subject variation in the effects of psychostimulants on cognitive functions.

### Ventral tegmental area/substantia nigra, pars compacta (VTA/SNc)

Thus, MPH diminishes negative connectivity between VTA/SNc and left MOG. While the prefrontal cortex receives both direct NE and DA projections, the occipital cortex receives scarce DA projections in the rat (Descarries et al., 1987; Lidow et al., 1991; Devoto et al., 2001). On the other hand, extra-cellular DA in the rat occipital cortex is only 37% lower than that in the mPFC (Devoto et al., 2003). In this same study, when an NE antagonist was injected into LC, NE, and DA levels in the occipital cortex fell 50 and 70%, respectively (Devoto et al., 2003). Conversely, a NE-agonist increased both occipital NE and DA (Devoto et al., 2004). Combined, these results suggest that occipital cortical DA may originate from co-release of DA and NE from LC neurons (Devoto et al., 2003, 2004).

It is plausible that MPH diminishes negative connectivity between VTA/SNc and the MOG as a result of complex interaction of NA and DA systems. Further, DA projections to cortex are more abundant in primates including humans than in rats (Björklund and Dunnett, 2007); for example, varying levels of DA-receptor mRNA have been found in the human occipital cortex, with D1 and D3 receptor transcripts being the most and least prominent, respectively (Meador-Woodruff et al., 1996). Further research should clarify this interaction and investigate its effect on behavior.

Many studies have implicated the MOG in ADHD. In diffusion tensor imaging (DTI), ADHD patients show reductions in trace (a measure of diffusion magnitude) in the left MOG (Alexander et al., 2007; Chaim et al., 2014). More specifically, children with ADHD inattentive subtype exhibit increased radial diffusivity (diffusion perpendicular to fiber direction) compared to healthy controls (Lei et al., 2014). Lower fractional anisotropy in the fronto-occipital fasciculus is correlated with greater inattention symptoms in ADHD adults (Shaw et al., 2014). Functional differences in the MOG are also associated with ADHD. Visual regions including the MOG show decreased nodal efficiency during a working memory task in ADHD based on a “small-world” regime (Xia et al., 2014). In activation likelihood estimation meta-analysis, the MOG is less likely to be active in ADHD patients during executive function compared to controls (Dickstein et al., 2006). Thus, one is tempted to speculate that the treatment effects of MPH in ADHD may at least in part be related to functional connectivity between the MOG and VTA/SNc.

MPH reduced positive connectivity between VTA/SNc and multiple areas of the dorsomedial cerebellum including the vermis, culmen, and medial hemispheres. The cerebellum is important to cognition and motor control, with distinct areal connections to motor and prefrontal cortices (Middleton and Strick, 2000). The medial cerebellar cortex and dorsal cerebellar nuclei are more involved in motor coordination than lateral cerebellar hemispheres and ventrolateral cerebellar nuclei, which are more involved in learning and cognition (Jueptner et al., 1997a,b). Thus, the reduction in VTA/SNc positive connectivity to the vermis, culmen, and medial cerebellum may suggest a damping effect of MPH on motor activity.

Acute stimulation of the VTA/SNc increases cFOS immunoreactivity, an indirect measure of brain activity, in the dorsal cerebellum, and chronic DA antagonism decreased cFOS in the dorsal vermis while increasing it in the dorsal cerebellar hemisphere (Herrera-Meza et al., 2014). Administration of dextroamphetamine, a competitive DA agonist, increases activity in the dorsomedial cerebellum (Ernst et al., 1997; Schouw et al., 2013). While studies have shown a relative lack of DA in the cerebellum compared to the basal ganglia (Wagner et al., 1983; Martres et al., 1985; Jucaite et al., 2006), other work has uncovered anatomical connections between VTA and cerebellum (Ikai et al., 1992) as well as tyrosine hydroxylase- and dopamine transporter-immunoreactive axons (Melchitzky and Lewis, 2000), DA D5 receptors (Khan et al., 2000), and DA neurons (Hurley et al., 2003), supporting potential actions of the MPH in the cerebellum.

Cerebellum has been implicated in catecholaminergic dysfunction. Mice generated with decreased levels of monoamine oxidase A (MAO-A), an enzyme integral to maintaining normal catecholamine levels, exhibit smaller cerebellums, decreased Purkinje cell count and dendritic density, and vermal hypoplasia (Alzghoul et al., 2012). MAO-A/B knockout mice also show enhanced eye-blink conditioning, which depends on cerebellar integrity, suggesting another critical cerebellar process contingent on catecholaminergic signaling (Singh et al., 2013).

The link between catecholaminergic function and the cerebellum may lead to new insights in treating motor disorders like PD. DA signaling from the striatum, as well as input from the cerebellum, influences the plasticity of the PMC during motor learning, a process known to be abnormal in PD (Kishore et al., 2014). Also, a model for the dyskinesia symptoms caused by levodopa, a common treatment for PD, implicates dysfunctions in cerebellar modulation of sensory projections to the PMC (Kishore et al., 2014; Kishore and Popa, 2014). In the latter hypothesis DA regulation is critical to proper cerebellar and motor function as a whole, and supports the influence of MPH on cerebellar connectivity.

MPH increases cerebellar activity in both ADHD adults and children (Epstein et al., 2007), and restores cerebellar response during successful response inhibition (Rubia et al., 2011). A T2 relaxometry study, which uses T2 relaxation time to estimate cerebral blood flow, also suggests a normalizing effect of MPH on cerebellar activity in ADHD, with hyperactive and non-hyperactive subjects, respectively, exhibiting an increase and decrease in T2 relaxation time under MPH (Anderson et al., 2002). In a non-ADHD population, MPH significantly increases metabolic activity in the cerebellum of cocaine addicts, and D2 receptor availabilities are significantly correlated with the increase in metabolism (Volkow et al., 1999). These results again support our finding of significant interaction between MPH and VTA/SNc-cerebellum connectivity. With the link of the cerebellum to execution of learned motor tasks (Jueptner et al., 1997a), as well as memory (Imamizu et al., 2000) and recall (Shadmehr and Holcomb, 1997) of motor skills, MPH's effect may underlie reductions in hyperactivity in ADHD (Cohen et al., 1971), and/or represent a more global shift to support cognitive over motor functions.

### Limitations and conclusions

Several issues should be considered. First, this observational study is based on a between-subjects design and did not involve a placebo control; thus, the placebo effect may confound the results. Further, we did not control for individual differences such as personality traits and pharmacokinetics, which may underlie variability of the effects of MPH. Thus, although the MPH and no-MPH groups are also individually matched in age and gender, the current results should be considered as preliminary and require replication in future work with a within-subject design. Second, the LC seed is very small. Although our recent study examined and negated the influence of physiological signals on BOLD activity and functional connectivity of the LC (Zhang et al., 2015), more studies with higher field magnet are needed to provide the spatial resolution needed to confirm the current findings. Third, we did not include any assessment of cognitive or motor performance, so the functional implications of our findings need to be reconsidered in follow-up work.

In summary, MPH had varying effects on the functional connectivity of BNM, LC, and VTA/SN. MPH reversed negative BNM connectivity with bilateral precentral gyrus, in accord with its effects on motor control and learning in ADHD and PD. MPH decreased positive connectivity between LC and cerebellum, which may underlie priming for cognitive over motor processing. MPH increased connectivity between LC and hippocampus, a change that may underlie reported improvements in memory. MPH eliminated or nearly eliminated connectivity of VTA/SNc with the cerebellum, putamen and left MOG, suggesting a DA mechanism of its effects on cognitive motor processing and visual attention. However, the current findings are obtained in healthy individuals and may not readily generalize to neuropsychiatric populations.

## Author contributions

All authors made substantial contributions to the conception or design of the work, data acquisition and analysis, interpretation of data; and drafting or revising the work for publication. All authors approved the final version and agreed to be accountable for the whole contents of the work.

## Funding

This study is supported by NIH grants DA026990, DA023248, AA021449, NS23945 (LZ), T32 NS07224 (OMF), and K25DA040032 (SZ) as well as the Peter McManus Trust, and conducted partly as a senior thesis project (RK) in the Department of Psychology at Yale University. The content is solely the responsibility of the authors and does not necessarily represent the official views of the National Institute of Drug Abuse or the National Institutes of Health.

### Conflict of interest statement

The authors declare that the research was conducted in the absence of any commercial or financial relationships that could be construed as a potential conflict of interest.
